# Physicochemical characterization and antioxidant activity of Palestinian honey samples

**DOI:** 10.1002/fsn3.754

**Published:** 2018-09-11

**Authors:** Hamada Imtara, Youssef Elamine, Badiâa Lyoussi

**Affiliations:** ^1^ Faculty of Sciences Laboratory of Physiology, Pharmacology and Environmental Health Dhar El Mehraz BP 1796 Atlas University Sidi Mohamed Ben Abdallah Fez Morocco

**Keywords:** antioxidant activity, honey, hydroxymethylfurfural, Palestine, physicochemical

## Abstract

Physicochemical characteristics, main minerals, and antioxidant activity were determined for Palestinian honey samples belonging to different floral and geographical origins. The features of the analyzed samples were within the established international standards for honey quality control. One clear exception was the hydroxymethylfurfural (HMF) of the Ziziphus sample purchased from the Jericho region, which is the lowest city in the word characterized by a hot desert climate. The observed HMF value was 81.86 ± 2.64 mg/kg being two folds the maximum allowed in honey samples (40 mg/kg). As a second objective of the present work, the parameters were divided into two groups with different discriminatory power. The assessed physicochemical parameters, and the antioxidant activities, specific to the botanical origin discrimination, were used to run the first PCA. A strong correlation could be seen between the bioactive compounds and the antioxidant activities despite the geographical origin of the samples. Thyme and Ziziphus samples were the best samples, while citrus sample presented the lowest activity. Regarding the geographical discrimination, Ash and mineral contents in addition to the electrical conductivity were used. The output PCA conserved high represent ability of the data in the two‐first components being 82.72% and 9.60%. A little discrimination between the samples produced in the north and those produced in the south of the country, but it was not perfect. The intervention of the botanical variability could be the reason.

## 1 INTRODUCTION

In Palestine, honey constituted a source of sugar for a long time. Ceramic investigation revealed the presence of an extensive beekeeping activity, and honey production as a source of sugar, during the Mamluk and Ottoman periods (Taxel, 2006). According to the Mediterranean Beekeepers Association, honey is an important economic and medical fortune and Palestine produces about 1,250 tons of honey per year (“Ramallah Beekeepers Cooperative,” 2015). The production of honey requires an attention from the scientific community for the characterization and standards establishing. In addition, despite its small geographical area, Palestine has a high diversity of plants and great variation in topography and climate from the arid to humid (CBD, 2015). 2,000 plants species are described, from which 393 species constitute a big potential of melliferous sources (Albaba, 2015).

As a natural product made by honeybees form the nectar or the sweet juice of different parts of the flowering plants, honey is a supersaturated sugar solution in combination with minerals, enzymes, vitamins, flavoring organic compounds, free amino acids, and numerous volatile compounds (Gorjanović et al., 2013; Kayode & Oyeyemi, 2014). The verity of its sources subject its composition to high variability, which require standardization procedures for customer's protection (Albaba, 2015). In addition to the floral origin, other factors may be determinant in the final quality of honey such as the geographical and climate characteristics as well as the processing and storage conditions (Aazza, Lyoussi, Antunes, & Miguel, 2013; Imtara, Elamine, & Lyoussi, 2018).

The sensorial, chemical, physical, and microbiological characteristics of honey determine its quality (Khalil et al., 2012). EC Directive 2001/110 has specified the criteria for ensuring honey quality (European Community, 2004), concerning mainly, the electrical conductivity, moisture content, reducing and non‐reducing sugars, pH, free acidity, diastase activity, ash content, HMF, and protein content.

At the best of author's knowledge, no previous study aimed a detailed characterization of commercialized honey samples in Palestine. Therefore, the main aim of the present work was to illustrate the quality characteristics of honey samples purchased from different areas of Palestine. The samples belong to different botanical origin and were characterized using a panel of known physicochemical parameters. In addition, ABTS, DPPH, iron reducing ability, and phosphomolybdenum reducing ability were assessed for the estimation of honey antioxidant activities. The entire data were used to study the correlations between the evaluated parameters, and to run the principal component analysis (PCA) for the discrimination of honey samples. The results were compared to the established quality standards, and to the reported honey samples belonging to the same botanical origin when it is possible.

## 2 MATERIAL AND METHODS

Ten local Palestinian honey samples were purchased from beekeeper, stored at room temperature (22–24°C) in airtight plastic containers until analysis, and labeled based on the commercial descriptions (Table 1). Visually, no sample of honey showed signs of fermentation or granulation before the characterization. Each assay was performed in triplicate, and the results were expressed as means ± *SD*.

**Table 1:** Honey samples IDs and their botanic, geographic origins, and harvest year

Code	Arabic name	English name	Scientific name	Location	Year of harvest
S 1	Zohif	Thyme	*Coridothymus capitatus*	Al‐Khalil	2014
S 2	Rabat	Hairy fleabane	*Conyza bonariensis*	Salfeet	2014
S 3	Multifloral	Multifloral	*Multifloral*	Tubas	2014
S 4	Limon	Citrus	*Citrus limon*	Jenin	2014
S 5	Multifloral	Multifloral	*Multifloral*	Ramallah	2014
S 6	Rabat	Hairy fleabane	*C. bonariensis*	Nablus	2014
S 7	Rabat	Hairy fleabane	*C. bonariensis*	Qalqilya	2014
S 8	Morar	Cornflower	*Centaureadumulosa Boiss*	Nablus	2014
S 9	Jabali	Rocky Mountain	*Valeriana tuberosa*	Bethlehem	2014
S 10	Sader	Ziziphus	*Ziziphusspina‐christi*	Jericho	2014

### 2.1 pH, free acidity, moisture, electrical conductivity, ash and proline content

The standardized methods of the International Honey Commission (IHC) were followed to assess the mentioned parameters (Bogdanov, 2009).

### 2.2 Colour and melanoidins content estimations

The color was determined with a spectrophotometer by reading the absorbance of honey aqueous solutions at 635 nm (50% W/V) (Naab, Tamame, & Caccavari, 2008). The obtained absorbance was used to estimate the color in mmPfund following the algorithm: mmPfund = −38.7 + 371.39 × absorbance.

Honey color was also determined spectrophotometrically by measuring the difference between two net absorbances at 560 and 720 nm. Melanoidins content was estimated based on the browning index (net absorbance at A450‐A720) (Brudzynski & Miotto, 2011), and the results were expressed as absorption units (AU).

### 2.3 Hydroxymethylfurfural

The HMF content was determined followed the spectrophotometric procedure described in (Elmer, 1979).

### 2.4 Determination of mineral elements

A 5 ml of nitric acid 0.1 M were added to the ashes, and the mixture was stirred on a heating plate to almost complete dryness. Then, 10 ml of the same acid was added for the solubilization and made up to 25 ml with distilled water. The mineral components were determined by atomic absorption spectrometry (Silva, Videira, Monteiro, Valentão, & Andrade, 2009).

### 2.5 Estimation of total antioxidant capacity by phosphomolybdate assay (TAC)

The TAC was estimated by the phosphomolybdenum method according to the reported procedure (Prieto, Pineda, & Aguilar, 1999). The assay is based on the reduction of Mo (VI)–Mo (V) by the honey solutions and subsequent formation of a green phosphate/Mo (V) complex in acid medium. Briefly, 25 μl of honey solution was combined with 1 ml of reagent solution (0.6 M sulfuric acid, 28‐mM sodium phosphate and 4‐mM ammonium molybdate). The tubes containing the reacting medium were capped and incubated in a boiling water bath at 95°C for 90 min. After cooling to room temperature, the absorbance of the solution was measured at 695 nm. The TAC of each sample was expressed as mg of ascorbic acid equivalent/g (mgAAE/g).

### 2.6 Total polyphenolic content

The total polyphenolic content estimation was based on the Folin–Ciocalteu protocol (Singleton & Rossi, 1964). A volume of 100 μl of honey solution was mixed with the 0.5 ml of Folin–Ciocalteu phenol reagent (1:10 dilution with distilled water) and 400 μl of 0.7 M Na_2_CO_3_ solution. The reaction mixture was incubated for 2 hr and in darkness; and the absorbance was measured at 760 nm. The total content of each sample was expressed as mg gallic acid equivalent/100 g (mg GAE/100 g).

### 2.7 Total flavone and flavonol content

The evaluation of flavone and flavonol content was carried out as previously described (Miguel, Nunes, Dandlen, Cavaco, & Antunes, 2014). Briefly, a volume of 500 μl of honey dilution was mixed with the 500 μl of AlCl_3_ (5%) and incubated for 1 hr at room temperature. The absorbance of the resulting solution was measured at 420 nm. The calibration curve was performed using quercetin dissolved in 96% ethanol with serial dilutions. Total flavone and flavonol content of each sample was expressed as the quercetin equivalent/100 g (QE/100 g).

### 2.8 Determination of free radical scavenging activity by DPPH method

0.1 mM DPPH solution was prepared and added in a volume of 825 to 150 μl of honey solutions diluted in series. The mixture was vigorously shacked and then incubated at room temperature for 1 hr in the dark. The absorbance at 517 nm was measured, and the scavenging activity of the DPPH radical was expressed as inhibition percentage using the following equation: % Inhibition = ([control absorbance − sample absorbance]/control absorbance) × 100. Butylatedhydroxytoluene (BHT) was used as positive control. The concentration providing 50% radical inhibition (IC_50_) was calculated from the graph of inhibition percentage plotted against honey concentrations (Brand‐Williams, Cuvelier, & Berset, 1995).

### 2.9 Capacity for scavenging 2,2′‐azino‐bis (3‐ethylbenzothiazoline‐6‐sulphonic acid) (ABTS)

The determination of ABTS radical scavenging ability was carried out as described previously (Miguel, 2009). The ABTS solution was made by mixing a volume of 7 mM of aqueous 2,2′‐azino‐bis(3‐ethylbenzothiazoline‐6‐sulphonic acid) (ABTS) and an equal squantity of 2.4 mM K_2_S_2_O_8_ followed by an incubation for 16 hr in the dark to produce cationic ABTS. A volume (825 μl) of ABTS solution added to 150 μl of honey solutions diluted in series. The absorbance was measured at 517 nm, and the determination IC_50_ was similar to the methodology described in the DPPH section. Trolox was used as positive control.

### 2.10 Reducing power assay (Iron reducing activity)

A volume of 150 μl of various honey dilutions was added to 200 μl of 0.2 M potassium buffer (pH 6.6) and 200 μl of potassium hexacyano ferrate (1% w/v). The mixture was vortexes and incubated for 20 min at 50°C, followed by the addition of 200 μl of trichloroacetic acid (10% w/v), 600 μl of distilled water, and 120 μl of ferric chloride (0.1%, w/v). The absorbance of the mixture was measured at 700 nm. The honey concentration providing 50% inhibition (IC_50_) was calculated from the graph of optical density (Oyaizu, 1986).

### 2.11 Statistical analysis

The statistical analysis were performed by ANOVA through the GraphPad Prism 6 program and using the Tukey's post hoc test at *p* < 0.05. Correlations between phenol and flavonoid contents and antioxidant activity were achieved by Pearson correlation coefficient (*r*) at a significance level of 99% (*p* < 0.01). The data pre‐processing and the PCA were accomplished using MultBiplot64 running in MATLAB R2017a.

## 3 RESULTS AND DISCUSSION

### 3.1 Quality control analysis

The analyzed honey samples presented acidic pH values, between 3.66 ± 0.01 in S2 and 4.25 ± 0.01 in S4 (Table 2). Such values are within the range accepted for honey (Bogdanov, Ruoff, & Oddo, 2004), and were similar to those found in Algerian, Portuguese, and Morocco honeys (Aazza et al., 2013; Elamine et al., 2017; Khalil et al., 2012). The acid pH inhibits the growth of microorganisms (Terrab, Díez, & Heredia, 2002). The free acidity of honey can be explained by the presence of organic acids in equilibrium with lactones, esters, and some inorganic ions such as phosphate. A high acid value indicates the fermentation of sugars into organic acids (Abselami et al., 2018). None of these samples exceeded the permitted acidity limit (50 mEq/kg) indicating the absence of undesirable fermentation process (European Community, 2004). The maximum value was seen in sample S5 (32.67 mEq/kg), while sample S4 presented the minimal value (11.67 mEq/kg) (Table 2).

**Table 2:** Physicochemical characterization of the analyzed samples

Code	pH	Free acidity (mEq/kg)	Moisture (%)	Conductivity (μS/cm)	Ash (%)	Pfund scale (mm)	Color discription	Color (A560–A720)	Melanoidin (A450–A720)	HMF (mg/kg)	Proline (mg/kg)
S 1	4.23 ± 0.01^a^	18.67 ± 1.15^ab^	19.27 ± 0.12^a^	449.67 ± 3.06^a^	0.208 ± 0.01^a^	82.064 ± 4.33^a^	Light amber	0.195 ± 0.010^a^	0.504 ± 0.010^a^	30.05 ± 0.09^e^	343.93 ± 20.96^abcd^
S 2	3.66 ± 0.01^cd^	22.17 ± 0.29^ab^	18.60 ± 0.46^a^	317.83 ± 56.44^abc^	0.065 ± 0.01^d^	65.207 ± 3.84^ab^	Light amber	0.074 ± 0.017^c^	0.110 ± 0.012^bc^	29.47 ± 0.15^e^	482.53 ± 5.30^ab^
S 3	4.12 ± 0.01^b^	17.92 ± 1.01^ab^	17.53 ± 0.58^b^	417.67 ± 2.08^ab^	0.102 ± 0.01^abc^	45.579 ± 4.66^abc^	Light extra amber	0.111 ± 0.003^ab^	0.229 ± 0.010^b^	22.91 ± 0.22^f^	330.82 ± 14.62^abc^
S 4	4.25 ± 0.01^a^	11.67 ± 1.04^c^	16.20 ± 0.1^c^	261.20 ± 1.15^abc^	0.150 ± 0.01^b^	49.127 ± 8.48^abc^	Light extra amber	0.091 ± 0.030^c^	0.130 ± 0.047^bc^	16.02 ± 0.12 ^g^	258.94 ± 5.32^e^
S 5	3.78 ± 0.01^cd^	32.67 ± 0.29^a^	18.87 ± 0.46^a^	432 ± 3^ab^	0.104 ± 0.02^abc^	75.077 ± 2.17^a^	Light amber	0.077 ± 0.022^c^	0.218 ± 0.025^b^	39.46 ± 0.37^b^	471.52 ± 15.98^ab^
S 6	3.82 ± 0.01^c^	27.33 ± 0.76^a^	19 ± 0.2^a^	386.33 ± 2.31^ab^	0.173 ± 0.02^a^	63.433 ± 1.83^ab^	Light amber	0.113 ± 0.009^ab^	0.156 ± 0.007^b^	10.16 ± 0.53 ^h^	368.51 ± 3.46^abc^
S 7	3.87 ± 0.01^c^	31.83 ± 1.15^a^	19.13 ± 0.12^a^	533.67 ± 3.06^a^	0.152 ± 0.01^b^	62.768 ± 2.27^ab^	Light amber	0.115 ± 0.020^ab^	0.185 ± 0.017^b^	41.28 ± 0.37^b^	720.87 ± 5.18^a^
S 8	3.73 ± 0.01^cd^	29.67 ± 0.58^a^	17.07 ± 0.23^b^	379 ± 2.65^ab^	0.119 ± 0.01^ab^	76.186 ± 8.98^a^	Light amber	0.115 ± 0.028^ab^	0.252 ± 0.020^b^	37.09 ± 0.19^bc^	473.87 ± 5.27^a^
S 9	3.84 ± 0.01^c^	22.08 ± 0.95^ab^	18.73 ± 0.64	346.67 ± 2.08^ab^	0.079 ± 0.01^d^	73.746 ± 7.06^ab^	Light amber	0.150 ± 0.022^a^	0.204 ± 0.022^b^	33.22 ± 0.07^d^	229.44 ± 3.24^e^
S 10	3.88 ± 0.01^c^	29.17 ± 2.57^a^	20.20 ± 0.4^a^	482.67 ± 12.74^a^	0.178 ± 0.05^a^	100.96 ± 2.27^a^	Amber	0.239 ± 0.012^a^	0.537 ± 0.012^a^	81.86 ± 2.64^a^	569.82 ± 10.80^a^

*Note*

Values in the same column followed by the same letter are not significant different (*p *<* *0.05) by the Tukey's multiple range test.

The moisture content of a honey sample depends on the environmental conditions and the manipulation by the beekeepers, which explain its usual year to year variations (Acquarone, Buera, & Elizalde, 2007). The moisture of the studied honey samples was within the standards (not more than 20%) (Codex Alimentarius Commission, 2001; European Community, 2004), except the Ziziphus honey (S10) with a moisture values of 20.2%. This value is similar to the Moroccan Ziziphus honey (Aazza, Lyoussi, Antunes, & Miguel, 2014) and higher than the Sudanese and Algerian Ziziphus honeys (Idris, Mariod, & Hamad, 2011; Zerrouk, Seijo, Escuredo, & Rodríguez‐Flores, 2018), which explain the governance of the environmental conditions on determining this parameter. 16.9% was the minimum value, seen in the case the sample S4. High moisture content allows the fermentation of honey by undesirable osmo‐tolerant yeasts and thus the formation of ethyl alcohol and carbon dioxide. In addition, ethyl alcohol can in turn oxidize to acetic acid and water giving a bitter taste to the honey (Chirife, Zamora, & Motto, 2006).

The Ash content of honey samples, determining the mineral richness and the resulting electrical conductivity are important parameters in determining the botanical origin of a honey sample (Aazza et al., 2013). In addition, the mentioned parameters serve as differentiating features between nectar and honeydew honeys (Louveaux, 1959). The Ash content of the analyzed samples was between 0.065 ± 0.01% (S2) and 0.208 ± 0.01% (S1), being below 0.6%, the determined threshold for honey samples (Codex Alimentarius Commission, 2001). The results of our study show that the electrical conductivity values of the honey samples vary between 261.2 ± 1.15 μS/cm in sample S4 (Citrus) and a maximum of 533.67 ± 3.06 μS/cm in sample S7 (hairy fleabane) (Table 2). The electrical conductivity measures the ionizable organic and inorganic substances and is not suitable to surpass 800 μS/cm, from a quality control point of view (Codex Alimentarius Commission, 2001). The values were similar to other Palestinian honey samples (Imtara et al., 2018), published earlier by the same group for other purposes, and to other samples from different botanical and geographical origins (Aazza et al., 2013; Elamine et al., 2017; Imtara et al., 2018). Other criteria used to determine the nutritional value of honey, with direct relation with the ash content and electrical conductivity is the mineral content (Table 3). Quantitatively speaking, potassium was the most important mineral among the eight evaluated elements, with prevalence in the sample S7 (495.48 ± 1.94 mg/kg), while S2 had the lowest value (190.23 ± 0.74 mg/kg). Sodium and calcium came in second place after potassium. The samples S8 had the highest sodium and calcium concentrations being present at levels of 196.51 ± 0.36 and 138.41 ± 0.73 mg/kg, successively. In addition, the trace minerals of Fe, Zn, Cu, and Pd were detected in all honey samples at low concentrations (Table 3). All values found in the samples were within the ranges reported for honeys from other study (Aazza et al., 2013; Fernández‐Torres et al., 2005; Imtara et al., 2018). The mineral composition of honey samples is also a potential indicator of its geographical origin, as well as a biomarker of possible pollution by toxic metals (Alves, Ramos, Gonçalves, Bernardo, & Mendes, 2013; Pohl, 2009).

**Table 3:** Mineral content in the analyzed honey samples

Code	K (mg/kg)	Na (mg/kg)	Ca (mg/kg)	Mg (mg/kg)	Fe (mg/kg)	Zn (mg/kg)	Cu (mg/kg)	Pd (mg/kg)
S 1	471.63 ± 0.55^c^	68.47 ± 0.49^e^	96.95 ± 0.35^b^	22.98 ± 0.11^f^	4.24 ± 0.03^b^	25.20 ± 1.52^a^	0.70 ± 0.03^f^	0.56 ± 0.01^f^
S 2	190.23 ± 0.74^j^	56.42 ± 0.68^f^	65.48 ± 0.56^h^	23.29 ± 0.15^f^	2.25 ± 0.05^bcde^	2.23 ± 0.03^c^	0.83 ± 0.01^e^	0.57 ± 0.01^f^
S 3	250.94 ± 0.39^h^	74.06 ± 0.02^d^	98.65 ± 0.15^b^	39.36 ± 0.22^b^	8.75 ± 1.36^a^	0.13 ± 0.01^cde^	0.79 ± 0.01^e^	0.94 ± 0.01^a^
S 4	203.92 ± 0.17^i^	35.19 ± 0.93^h^	92.01 ± 0.60^d^	30.26 ± 0.51^e^	3.80 ± 1.39^bc^	1.27 ± 0.01^c^	0.95 ± 0.01^d^	0.79 ± 0.01^d^
S 5	435.93 ± 0.16^d^	51.65 ± 1.46^g^	74.98 ± 2.07^e^	32.00 ± 0.15^d^	2.49 ± 0.97^bcd^	1.15 ± 0.01^c^	0.66 ± 0.01^f^	0.57 ± 0.01^f^
S 6	441.32 ± 0.90^e^	73.88 ± 0.35^d^	71.26 ± 0.15^f^	22.65 ± 0.11^f^	5.47 ± 1.52^b^	1.92 ± 0.02^c^	1.22 ± 0.02^a^	0.89 ± 0.02^b^
S 7	495.48 ± 1.94^a^	69.56 ± 1.59^e^	68.01 ± 0.08^g^	33.95 ± 0.28^c^	3.56 ± 0.23^b^	2.35 ± 0.04^c^	0.96 ± 0.01^d^	0.83 ± 0.01^c^
S 8	322.54 ± 0.86^f^	196.51 ± 0.36^a^	138.41 ± 0.73^a^	54.15 ± 0.91^a^	4.77 ± 0.83^b^	13.76 ± 0.77^b^	1.15 ± 0.02^b^	0.74 ± 0.01^e^
S 9	238.85 ± 1.01^g^	110.77 ± 1.38^c^	64.49 ± 0.05^h^	20.48 ± 0.17^g^	4.29 ± 0.69^b^	0.80 ± 0.01^cd^	0.61 ± 0.01^g^	0.51 ± 0.01^g^
S 10	476.40 ± 1.51^b^	115.04 ± 0.04^b^	94.56 ± 0.39^c^	34.48 ± 0.17^c^	3.93 ± 1.20^b^	2.48 ± 0.04^c^	1.02 ± 0.01^c^	0.84 ± 0.02^c^

*Note*

Values in the same column followed by the same letter are not significant different (*p *<* *0.05) by the Tukey's multiple range test.

The correlation matrix of some analyzed physicochemical parameters and the mineral compositions are illustrated in Table 6. Ash content has a strong positive correlation with potassium (*r *=* *0.708, *p *<* *0.05), explaining the prevalence of potassium in all analyzed honey samples (Table 3). The same correlation was reported previously (Hazali et al., 2017). As the Ash content determines the electrical conductivity of honeys (Guler, Bakan, Nisbet, & Yavuz, 2007), a strong positive correlation was also seen between the potassium levels and the electrical conductivity (*r *=* *0.847, *p *<* *0.001).

Proline, an essential free amino acid used for quality control of honey samples (Paramás, Bárez, Marcos, García‐Villanova, & Sánchez, 2006). Values below 180 mg/100 g may indicate the none ripeness of a honey sample and/or adulteration (Bogdanov et al., 1999). None of the analyzed samples presented less amount with the maximum proline content found in hairy fleabane sample (S7) (720.87 ± 5.18 mg/kg) coming from Qalqilya. This value was three folds higher than the minimum value seen in Rocky Mountain honey sample (S9) coming from Bethlehem (229.44 ± 3.24 mg/kg) (Table 2).

Regarding the color classification, multifloral honey from tubas and Citrus honey presented light extra amber color, Ziziphus honey had an amber color, while the remaining samples presented light amber colors. The color of honey is influenced by various factors, including mineral content (Gomes, Dias, Moreira, Rodrigues, & Estevinho, 2010). The estimations of melanoidin, which are heterogeneous polymers of high molecular weight of brown color, have a very important role in discriminating the botanical origin of honey samples (Da Silva, Gauche, Gonzaga, Costa, & Fett, 2016). They are formed when sugars and amino acids combine (through the Maillard reaction) at high temperatures and low water activity (Amarowicz, 2009). In this way, the darkness of some honeys can be mainly attributed to the melanoidin which may indicate long periods of storage and/or honey heating processes (Borrelli, Visconti, Mennella, Anese, & Fogliano, 2002; Martins & Jongen, 2001).

As honey color is also governed by the polyphenolics and melanoidin content (Aazza et al., 2013, 2014). A strong positive correlation was obtained between color, from one side, and melanoidin and polyphenols, from the other side, with r values of 0.599 (*p* < 0.05), 0.911 (*p* < 0.001), respectively. The color of the analyzed samples correlated also, with high significance (*p* < 0.001), the amounts of flavones and flavonol (*r* = 0.893).

Regarding HMF level, another sensitive parameter for heating and storage conditions (Aazza et al., 2014), eight of the analyzed samples presented levels less than the maximum established by international standards (<40 mg/kg) (Codex Alimentarius Commission, 2001; European Community, 2004). In contrast, honey sample S7 showed slight higher value in comparison to the norms, while S10 originating from Jericho presented a very high amount of HMF being 81.86 ± 2.64 mg/kg. The last value could be explained by the semi‐arid microclimate characterized by mild to warm winter and hot dry summer known in Jericho region. Also the storage conditions in Jordan Valley (Jericho) characterized by hot desert climate may be key influencing factor to change the honey quality and can therefore explain the high value of HMF (Awad, Rząd, & Busse, 2014). It has be mentioned that the HMF content may be influenced by the temperature, storage conditions, the physicochemical properties of honey (pH, acidity, moisture, etc.) and by the concentrations of metallic ions such as manganese, zinc, magnesium, and iron (II) presenting honey (Shapla, Solayman, Alam, Khalil, & Gan, 2018).

### 3.2 Bioactive compounds and antioxidant activity

The results of this section are illustrated in Table 5. Regarding the bioactive compounds, namely polyphenolic, flavones and flavanol, a great variability was seen among the analyzed samples, suggestion the intervention of the reported floral influence (Fernández‐Torres et al., 2005), as they originate from different botanical sources. The lowest polyphenolic content value was obtained in hairy fleabane (S2) from Salfeet (26.96 ± 0.71 mg/100 g), while the highest value was obtained in thyme honey (S1) from Al‐Khalil (70.73 ± 0.71 mg/100 g). This value is similar to that found in thyme honey from Morocco (Aazza et al., 2014). The highest content of flavones and flavanol was found in S10 honey with a value of 8.23 ± 0.59 mg QE/100 g, while a minimum value of 0.18 ± 0.04 mg QE/100 g was seen in samples S3 (Table 5).

The ability of the analyzed samples to scavenge DPPH free radicals, expressed as IC_50 _mg/ml, was also evaluated. The lowest IC_50_ was seen in the case of samples S1 and S10, being, so, the most efficient samples regarding the DPPH free radicals scavenging. Their values were 9.04 ± 0.68 and 14.81 ± 2.16 mg/ml, respectively. Both samples presented the highest values of polyphenolic compounds (70.73 ± 0.84 mg GAE/100 g and 63.24 ± 0.60 mg GAE/100 g), flavones and flavonol contents (5.09 ± 0.05 mg QE/100 gand 8.23 ± 0.59 mg QE/100 g) among the analyzed. Such relation could be seen clearly through the negative correlation between the mentioned bioactive compounds and the DPPH IC_50_ (Table 4). Both *r* values were negative, but the significant level was reached only in the case of flavones and flavonol contents (*r* = −0.738; *p* < 0.05). Similar results, and correlation behavior were obtained by other groups when analyzing honeys samples from different botanical and geographical origins (Bertoncelj, Doberšek, Jamnik, & Golob, 2007; Khalil et al., 2012). BHT was used as positive control with a very low IC_50_ in comparison to honey samples (0.009 ± 0.0001 mg/ml).

**Table 4:** Pearson correlation coefficients among compounds and antioxidant activity

	Phenols	Flavones and flavonol	Antioxidantcapacity	Proline	Color	Melanoidin	DPPH	ABTS	Reducing power
Phenols	1	0.637*	0.007	−0.057	0.599*	0.643*	−0.082	−0.619*	−0.878***
Flavones and flavonol	0.637*	1	−0.654*	0.329	0.893***	0.942****	−0.738*	−0.504	−0.778**
Antioxidantcapacity	0.007	−0.654*	1	−0.296	−0.439	−0.526	0.727**	0.135	0.243
Proline	−0.057	0.329	−0.296	1	0.098	0.134	−0.458	−0.493	−0.234
Color	0.599*	0.893***	−0.439	0.098	1	0.911***	−0.500	−0.384	−0.681*
Melanoidin	0.643*	0.942****	−0.526	0.134	0.911***	1	−0.681*	−0.558*	−0.672*
DPPH	−0.082	−0.738*	0.727**	−0.458	−0.500	−0.681*	1	0.418	0.139
ABTS	−0.619*	−0.504	0.135	−0.493	−0.384	−0.558*	0.418	1	0.614*
Reducing power	−0.878***	−0.778**	0.243	−0.234	−0.681*	−0.672*	0.139	0.614*	1

****Correlation is significant at the *P < 0.0001*; ***Correlation is signification at the *P < *0.001; **Correlation is significant at the *P < *0.01;*Correlation is significant at the *P < *0.05.

Antioxidant activity was also assessed by the ABTS assay (Table 5), through which, we found that sample S1 was the most active presenting an IC_50_ of 3.26 ± 0.20 mg/ml. This results concordat the ones of the DPPH assay, which also explain the negative correlation between the IC_50_ of ABTS and the polyphenolic content (*r* = −0.619; *p* < 0.05). Honey sample S9 honey presented the highest IC_50_ 16.28 ± 1.25 mg/ml, being the less active sample. Trolox was used as positive control with IC_50_ of 0.019 ± 0.003 mg/ml.

**Table 5:** The content of phenols, flavones, and flavonols, total antioxidant capacity (TAC) and antioxidant activity (DPPH, ABTS and reducing power)

Code	Phenols (mg GAE/100 g)	Flavones and flavonol(mg QE/100 g)	TAC (mg AA/g)	DPPH (IC50 = mg/ml)	ABTS (IC50 = mg/ml)	Reducing power (IC50 = mg/ml)
S 1	70.73 ± 0.84^a^	5.09 ± 0.05^b^	84.18 ± 2.77	9.04 ± 0.68^f^	3.26 ± 0.20^d^	3.42 ± 0.04^bc^
S 2	26.96 ± 0.71^f^	−	87.04 ± 2.33	23.53 ± 0.81^e^	13.79 ± 0.32^ab^	5.03 ± 0.12^a^
S 3	27.72 ± 0.12^f^	0.18 ± 0.04^e^	111.97 ± 2.48	39.68 ± 0.05^d^	12.09 ± 0.10^ab^	5.32 ± 0.10^a^
S 4	54.78 ± 0.16^d^	0.27 ± 0.05^e^	117.98 ± 2.02	86.90 ± 1.69^a^	15.62 ± 0.61^a^	3.86 ± 0.06^bc^
S 5	41.64 ± 1.03^e^	0.69 ± 0.12^e^	94.85 ± 1.65	40.29 ± 0.98^d^	3.73 ± 0.51^d^	4.12 ± 0.07^b^
S 6	58.98 ± 1.32^c^	0.38 ± 0.10^e^	120.04 ± 1.59	75.47 ± 2.41^b^	3.77 ± 0.16^d^	3.85 ± 0.03^bc^
S 7	40.43 ± 0.70^e^	1.05 ± 0.09^d^	102.66 ± 1.15	38.86 ± 1.56^d^	5.53 ± 0.92^d^	4.00 ± 0.04^b^
S 8	44.06 ± 1.07^e^	2.90 ± 0.22^c^	81.18 ± 3.01	44.40 ± 0.06^d^	8.06 ± 0.13^c^	3.85 ± 0.03^bc^
S 9	30.82 ± 0.28^f^	0.51 ± 0.08^e^	87.29 ± 2.17	57.93 ± 2.72^c^	16.28 ± 0.25^a^	4.42 ± 0.18^b^
S 10	63.24 ± 0.60^b^	8.23 ± 0.59^a^	83.98 ± 1.35	14.81 ± 2.16^f^	3.60 ± 0.21^d^	2.84 ± 0.09^bc^
BHT	—	—	—	0.009 ± 0.0001 ^g^	—	—
Trolox	—	—	—	—	0.019 ± 0.003^e^	—
Ascorbicacid	—	—	—	—	—	0.003 ± 0.001^d^

*Note*

Values in the same column followed by the same letter are not significant different (*p *<* *0.05) by the Tukey's multiple range test.

**Table 6:** Pearson correlation coefficients among ash, conductivity, Pfund, melanoidin, and mineral elements

	K	Na	Ca	Mg	Fe	Zn	Cu	Pd
Ash	0.708*	−0.061	0.223	−0.060	0.067	0.510	0.376	0.338
Conductivity	0.847***	0.121	−0.011	0.189	0.076	0.182	0.021	0.235
Pfund	0.550*	0.446	0.145	0.003	−0.414	0.372	−0.003	−0.335
Melanoidin	0.567*	0.273	0.366	0.076	0.053	0.571*	−0.082	−0.033

***Correlation is signification at the *P < *0.001; **Correlation is significant at the *P < *0.01; *Correlation is significant at the *P < *0.05.

The reducing power of the studied honey samples is dose‐dependent. The results illustrated in Table 5 shows that the sample originating from Jericho was the most reducing sample, while Tubas honey had the lowest activity (5.32 ± 0.10 mg/ml). A possible effect of polyphenolic, flavones, and flavonol could be seen through the resulted positive correlation with the ability of samples to reduce the Fe^3+^ ions. The r values were *r* = −0.878 (*p* < 0.001) and *r* = −0.778 (*p* < 0.001) for polyphenols and flavonoids, successively.

For the three antioxidant activity, a possible role of Maillard reaction products, estimated by the melanoidin content, was clearly illustrated by the established positive correlation. In the present work, melanoidin showed significant (*p* < 0.05) correlations with the IC_50_ of DPPH, ABTS and the reducing power with *r* values of −0.681, −0.558, and −0.672, respectively.

Sample S6 presented the highest total antioxidant activity with a value of 120.03 ± 1.59 mg AAE/g, while sample S10 honey had the lowest activity (83.98 ± 1.35 mg AAE/g honey).

### 3.3 Multivariate analysis

To further understand the distribution of the analyzed samples, based on the assessed parameters, principal component analysis was used (PCA). PCA is known to be a good tool for information extraction from multivariate matrices and concentrate it in only few components (Bevilacqua, Bucci, Magrì, Magrì, & Nescatelli, 2013). The scores of the obtained components are then used to plot the data in an interpretable way.

**Figure 1:**
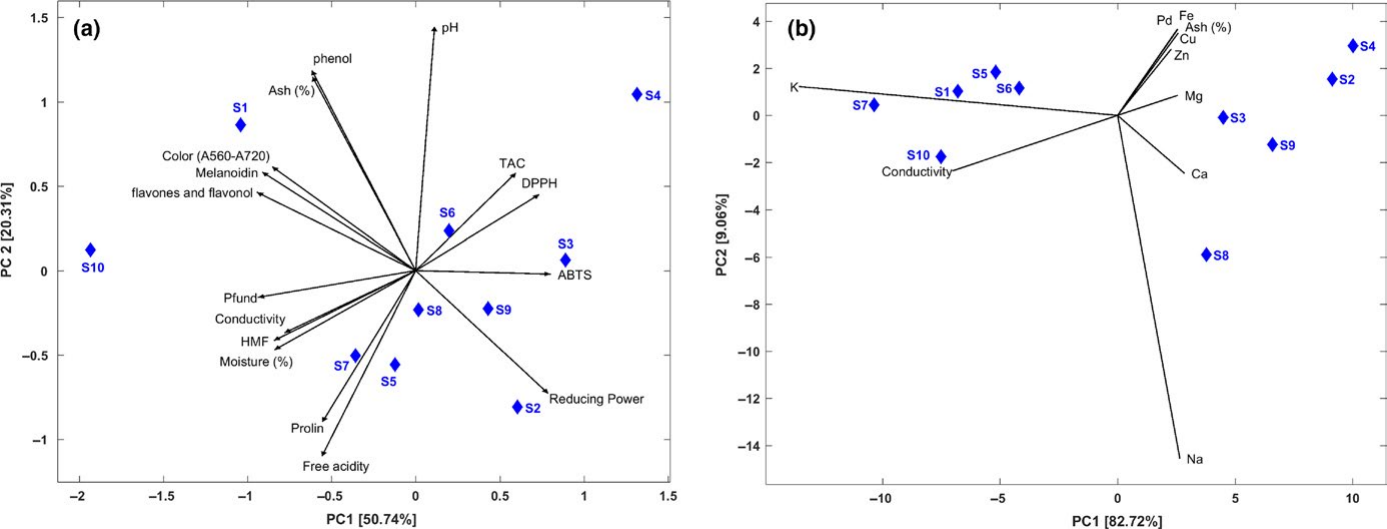
Principal component analysis of the assessed parameters. (a) PCA of the physicochemical parameters and the antioxidant activities. (b) PCA of the mineral content, ash, and electrical conductivity. K: potassium; Na: sodium; Ca: calcium; Mg: magnisum; Fe: iron; Zn: zinc; Cu: copper; Pd: palladium

In the present work, the evaluated parameters were divided into two main groups. The first group was formed by all parameters except the mineral content and was used as matrix to extract the information resulting from the botanical origin effect. The purpose was to cluster the Palestinian samples by their similarities in terms of physicochemical properties and antioxidant features. A second one was formed by the contents of minerals, the ash content, and the electrical conductivity. It is well established that this group of parameters, and besides being influenced by the botanical origin, it may indicate the geographical origins. It is then important to illustrate if there is a finger print characterizing samples produced in a specific Palestinian region. The results of both PCAs were illustrated in Figure 1a and b, successively.

Figure 1a describes the distribution of the honey samples based on the parameter illustrated as black narrows. The projection of each narrow on a given axis (component) reflects its represent ability/correlation with the same component. Honey samples were plotted as blue full diamonds. Considering the given data, 50.74% was conserved in the first principal component, which explained on the negative part color intensity, melanoidin, flavones, flavonol, and the slightly the polyphenols. Consequently, the first component correlated positively the IC_50_ of the assessed antioxidant activities. In addition, the same component correlated also the HMF, moisture contents and the electrical conductivity, but negatively. For the second component, 20.31% of the given data was retained and represented, mainly the pH, Ash content and polyphenolic content, in the positive part, and proline and free acidity in the negative part.

Thyme (S1) and Ziziphus (S10) honey samples shared the features regarding the bioactive compounds, and color intensity, being so the most antioxidant samples. This feature is already reported for both botanical origins (Aazza et al., 2014). The variability of the secondary plants, which may be specific to Palestine, seems to do not be significantly influencing, and both botanical origins seem to be a good option when honey antioxidant ability is desired. Honey samples labeled as hairy fleabane, Multifloral, Cornflower and Rocky Mountain shared the lowest pH values and high proline content in comparison to the remaining samples. Low pH value is a property that inhibits the growth of undesirable microbial entities. In addition, the authors of the present work reported that low pH values is favorable parameters when a synergetic affect with essential oils against microbial strains is targeted (Imtara et al., 2018).

Regarding the study of the geographical component in discriminating the analyzed honey samples, the given data (ash, mineral contents and electrical activity) was highly conserved in the first two principal components explaining 82.72% and 9.06%, respectively. Two main clusters could be distinguished regarding the first principal component. The first cluster was formed by S1, S5, S6, S7, and S10 and were characterized by high potassium content (the most abundant element among the assessed minerals [table]) and electrical conductivity. Among the mentioned honey samples, S6 was the only one harvested in the north part of Palestine. The second cluster was formed by S9 and S2 produced in the south of the country, and the remaining samples (S3, S4, and S8) provided from the north part. The five samples presented less potassium content and electrical conductivity and, relatively, less amounts of the remaining parameters.

The geographical clustering was not perfect, and exception could be seen. This may be due to the intervention of the botanic origin, as it is well documented to be also crucial in determining the mineral profile of honey samples (Karabagias et al., 2017). However, the discrimination of samples using the mineral profile, the ash content and the electrical conductivity was clearer than in the case of the parameters used in Figure 1a.

## 4 CONCLUSION

Except the high HMF content of the honey sample originating from Jericho, no abnormal feature could be highlighted about the analyzed Palestinian honey. As it is the lowest city above the sea level in the world, the resulting climate may be the reason of the HMF increase. Such a feature needs to be a central interest in a possible study with extended sampling to other botanical honey from the same region. This will discriminate the possible effect of the botanical source and highlight at which level extreme climate of the region affects the quality of the produced honey samples.

## CONFLICT OF INTEREST

The authors of the present work declare no conflicts of interest in relation to published information. The authors are responsible for the content and writing of the article.
